# Supplementary material for: “I Don’t Believe in Age; I Believe in Staying Enthusiastic”: An Exploratory Qualitative Study into Recruitment Strategies Stimulating Middle-Aged and Older Adults to Join Physical Activity Interventions
Source: Geriatrics (Basel). 2024 Jun 13;9(3):80. doi: 10.3390/geriatrics9030080 (PMC11202473; doi:10.3390/geriatrics9030080)
Supplement: Supplementary file 1 [file geriatrics-09-00080-s001.zip › 21032024 File S2 Codebook.pdf]

CODEBOEK

Coderen interviews met 50-plussers t.b.v. het implementatieproject beweeginterventies voor ouderen/Actief Plus

Onderzoeksvragen

1. Hoe kan deelname aan beweeginterventies voor 50-plussers c.q. deelname aan de beweeginterventie Actief Plus interventie worden bevorderd d.m.v. een op de doelgroep afgestemde aanpak van de werving:
    a. Welke demografische en/of persoonlijke variabelen van de doelgroep zijn het meest relevant om rekening mee te houden bij de werving van beweeginterventies voor 50-plussers?
    b. Welke eigenschappen van de wervingsboodschap spreekt de doelgroep het meest aan?
    c. Welke bron of combinatie van bronnen spreekt de doelgroep het meest aan?
    d. Welk kanaal of combinatie van kanalen spreekt de doelgroep het meest aan?
    e. Wat zijn volgens de doelgroep faciliterende en belemmerende determinanten m.b.t. het doelgedrag c.q. het aanmelden voor en meedoen aan de beweeginterventie Actief Plus, en hoe kunnen de belemmerende determinanten zo goed mogelijk verholpen worden?

Opnemen van gevraagde **achtergrondgegevens** in Atlas: waarschijnlijk via **documentgroups**

- Leeftijd
- Geslacht
- Opleidingsniveau
- Wel of niet een actieve beweger

1a. Welke demografische en/of persoonlijke variabelen van de doelgroep zijn het meest relevant om rekening mee te houden bij de werving van beweeginterventies voor 50-plussers?

1.Inputfactoren: Doelgroep/ontvanger

**Code**	**Input/ Communicatievariabele**	**Definitie**	**Instructie voor codering**	**Voorbeeld interviewvragen**
**1A**	**Demografische factoren:**	De perceptie van 50-plussers over de bevorderende en/of belemmerende werking van hun persoonlijke demografische factoren wanneer het gaat om het deelnemen aan een beweegprogramma.	Inclusie:<br>-Uitspraken die wel onder demografische variabelen vallen maar niet ondergebracht kunnen worden onder 1A1 t/m 1A4<br>-Uitspraken over het wel of niet opgevoed zijn in een omgeving waar bewegen de norm was<br><br>Exclusie:<br>-Uitspraken die onder 1A1 t/m 1A4 vallen<br>-Uitspraken die onder 1B t/m 1E vallen	• Vindt u het belangrijk dat het beweeggedrag van 50-plussers wordt gestimuleerd? Wat zijn redenen dat u dat belangrijk vindt of juist niet?<br>• Is beweeggedrag volgens u het belangrijkste of zijn er andere behoeften die meer prioriteit hebben om aangepast te worden bij 50-plussers?<br>• Denkt u dat het noodzakelijk is dat er beweegprogramma's worden ingezet om het beweeggedrag van 50-plussers te bevorderen?<br>• Wat zouden voor u overwegingen zijn om mee te

				doen aan een beweegprogramma?<br>• En wat zijn redenen die ervoor zorgen dat u niet wilt meedoen aan een beweegprogramma?
**1A1**	**Leeftijd**	De perceptie van 50-plussers over de bevorderende en/of belemmerende werking van hun leeftijd wanneer het gaat om het deelnemen aan een beweegprogramma.	Inclusie:<br>-Uitspraken over leeftijd in relatie tot beweeggedrag algemeen<br>-Uitspraken waarin de persoon aangeeft welk belang een beweegprogramma heeft voor iemand van hun leeftijd<br>-Uitspraken waarin de persoon aangeeft wel of niet te kunnen of willen meedoen aan een beweegprogramma gezien hun leeftijd<br>-Uitspraken over het eigen gevoel wanneer het gaat om hun leeftijd/leeftijdscategorie. Bv. vinden dat zij eigenlijk tot een jongere groep behoren<br>-Uitspraken over de zin of onzin van een beweeginterventie gericht op een specifieke doelgroep	• Voelt u zich aangesproken als het gaat over 50-plussers? Ziet u zichzelf als 50-plusser? Tot welke groep ziet u zich wel?<br>• Vindt u het aansprekender als het gaat over een brede doelgroep qua leeftijd voor beweegprogramma's of zou u het liever wat meer gericht op bepaalde leeftijdscategorieën zien?
**1A2**	**Geslacht**	De perceptie van 50-plussers over de bevorderende en/of belemmerende werking van hun geslacht wanneer het gaat om het deelnemen aan een beweegprogramma.	Inclusie:<br>-Uitspraken over geslachtsverschillen wanneer het gaat om beweeggedrag<br>-Uitspraken over het wel of niet kunnen of willen meedoen aan een beweegprogramma gezien hun geslacht<br>-Uitspraken over genderrollen die meedoen aan een beweegprogramma belemmeren of bevorderen (bv. ik kan niet voldoende bewegen vanwege de zorg over de kinderen of het huishouden)	

**1A3**	**Sociaaleconomische status**	De perceptie van 50-plussers over de bevorderende en/of belemmerende werking van hun sociaaleconomische status wanneer het gaat om het deelnemen aan een beweegprogramma.	Inclusie:<br>-Uitspraken over de kosten die bewegen met zich mee brengt en de mogelijkheid om die te dragen<br>-Uitspraken over opleidingsniveau of kennisniveau die het meedoen aan een beweegprogramma mogelijk beïnvloeden<br>-Uitspraken over de belemmerende of bevorderende factoren voor het meedoen aan een beweegprogramma vanwege hun beroep	
**1A4**	**Gezinssituatie**	De perceptie van 50-plussers over de bevorderende en/of belemmerende werking van hun gezinssituatie wanneer het gaat om het deelnemen aan een beweegprogramma.	Inclusie:<br>-Uitspraken over hoe de gezinssituatie beweeggedrag beïnvloed<br>-Uitspraken als niet kunnen meedoen aan een beweegprogramma door zorg voor kinderen of zorg voor een partner of ander familielid<br>-Uitspraken over hoe meedoen aan een beweegprogramma prettig kan zijn voor een alleenstaande door hun gezinssituatie<br><br>Exclusie:<br>-Uitspraken over sociaal contact welke niet direct gelinkt is aan de gezinssituatie -> **1F sociaal contact**	
**1B**	**Capaciteiten en motivatie:**	De perceptie van 50-plussers over hun eigen capaciteiten en motivatie wanneer het gaat om het deelnemen aan een beweegprogramma.	Inclusie:<br>-Uitspraken over angst om te vallen door gedachten of fysieke beperkingen, zoals een aandoening of weinig spierkracht<br>-Uitspraken met betrekking tot het wel of niet kunnen bewegen/ bewegingen kunnen uitvoeren om wat voor reden dan ook	• Op welke manieren zouden wij deelname aan het beweegprogramma voor u makkelijker kunnen maken?

			-Uitspraken over wel of niet willen bewegen<br>-Uitspraken over het bezitten van vaardigheden wanneer het gaat om beweeggedrag<br>-Uitspraken over de gelegenheid om te bewegen, nabijheid van voorzieningen en de mogelijkheid om hier te komen<br>-Uitspraken over wel of niet internetvaardig zijn<br><br><u>Exclusie:</u><br>-Uitspraken die aantonen dat de persoon over het algemeen angstig of negatief gestemd is, dus niet alleen bij beweging -> **Persoonlijkheid 1C**<br>-Uitspraken die aantonen dat de persoon over het algemeen geen behoefte heeft om dingen te proberen en te ervaren -> **Persoonlijkheid 1C**<br>-Uitspraken die aantonen dat de persoon motivatie/geen motivatie heeft op vlakken buiten beweeggedrag -> **Persoonlijkheid 1C**	
**1C**	**Persoonlijkheid:**	De perceptie van 50-plussers over eigenschappen van zichzelf die niet enkel gaan over beweeggedrag en opgevat kunnen worden als een persoonlijkheidskenmerk.	<u>Inclusie:</u><br>-Uitspraken over het openstaan voor/nieuwsgierigheid naar nieuwe ervaringen<br>-Uitspraken over het belang van routines<br>-Uitspraken over het belang van sociaal contact, behoefte aan gezelligheid of juist het tegenovergestelde<br>-Uitspraken over het vertrouwen in anderen in het algemeen, empathisch vermogen	

			-Uitspraken over zelfregulatie, impulsbeheersing, discipline, naleven van lange termijn doelen<br>-Uitspraken die de neiging tot angst, zelftwijfel en andere negatieve gevoelens.<br>-Uitspraken die anderzijds als persoonlijkheidskenmerk opgevat kunnen worden<br><br><u>Exclusie:</u><br>-Wanneer de uitspraken specifiek gaan over beweeggedrag en dus niet gegeneraliseerd kunnen worden als een persoonlijkheidskenmerk -> **Capaciteiten en motivatie 1B**	
**1D**	**Leefstijl:**	De perceptie van 50-plussers over de invloed van hun leefstijl wanneer het gaat om het deelnemen aan een beweegprogramma.	<u>Inclusie:</u><br>-Uitspraken over het passen van beweging in de eigen leefstijl<br>-Uitspraken over het belang, of juist niet, van een gezonde leefstijl<br>-Uitspraken over leefstijlfactoren die het beweeggedrag beïnvloeden (zoals slaapgedrag, stress, voeding, alcohol, roken etc.)	Bent u nu wel of niet een actieve beweger of bent u dat geweest?
**1E**	**Cultuur:**	De perceptie van 50-plussers op beweeggedrag die gelinkt is aan de eigen cultuur.	<u>Inclusie:</u><br>-Uitspraken over de (on)mogelijkheid om te sporten vanwege culturele of religieuze redenen.	

-

1b. Welke eigenschappen van de wervingsboodschap spreekt de doelgroep het meest aan?

2. **Inputfactoren: Bericht**

Code	Input/ Communicatievariabele	Definitie	Instructie voor codering	Voorbeeld vragen
**2A**	**Structuur**	De perceptie van de 50-plussers over de structuur van een wervingsbericht voor deelname aan een beweegprogramma	Inclusie:<br>-Uitspraken over de volgorde van de tekst<br>-Uitspraken over het stapsgewijs geven van informatie of juist niet<br>-Uitspraken over de grootte van het bericht<br>-Uitspraken over of de titel en kopjes goed bij de tekst passen of juist niet<br>-Uitspraken over de afsluiting van de tekst, vat deze de tekst duidelijk samen/moedigt aan om iets te doen of juist niet<br><br>Exclusie:<br>-Uitspraken die niet over de structuur van de tekst gaan maar over de inhoud, deze roepen emotie of een bepaalde beleving op -> **2B Soort argument**	• Wat is uw eerste indruk van deze berichten?
**2B**	**Soort argument**	De perceptie van de 50-plussers over het soort argument in het wervingsbericht voor deelname aan een beweegprogramma	Inclusie:<br>-Uitspraken over de argumenten waarop het wervingsbericht zich kan/moet richten wanneer het gaat om de voordelen van bewegen. Bijvoorbeeld het verminderen van depressieve klachten en andere mentale voordelen (cognitieve stabiliteit), leren doelen stellen en omgaan met moeilijke situaties (cognitieve groei),	• Wat is uw eerste indruk van deze berichten?<br>• Hoe aansprekend/ motiverend vindt u de berichten?<br>• Zijn er woorden die u aanspreken of juist niet? Wat is de reden dat u dat vindt?

			spanningsvermindering (affectieve stabiliteit), vergroten van zelfvertrouwen (affectieve groei)<br>-Uitspraken over behoeften waarop het wervingsbericht zich kan richten, zoals het bevorderen van sociaal contact d.m.v. bewegen<br>-Uitspraken over het geleverde bewijs dat er te weinig bewogen wordt<br>-Uitspraken over valkuilen waarop gelet moet worden als betutteling, vinger wijzen, te kritisch zijn waardoor de motivatie om mee te doen verloren raakt<br><br>Exclusie:<br>-Uitspraken naar aanleiding van het bericht die onder demografische variabelen vallen -> **1A Demografische variabelen**	• Zijn er zaken die niet in de berichten staan maar waarvan u wel vindt dat ze benoemd moeten worden? Welke zijn dit?<br>• Denkt u dat het beter is om te vertellen dat je van meer bewegen een betere conditie en meer kracht krijgt, of juist dat je door meer bewegen onafhankelijker in het leven kan staan?<br>• Welke informatie vindt u interessanter: Voldoende bewegen werkt beschermend tegen het ontwikkelen van verschillende ziekten als hart- en vaatziekten, beroerte, diabetes en verschillende en verschillende vormen van kanker. Of: Voldoende bewegen heeft een positief op de mentale gezondheid waarbij het gevoel van welzijn en kwaliteit van leven wordt vergroot, daarnaast kan voldoende bewegen het ontwikkelen van een depressie voorkomen.

				Wat is de reden dat u dat vindt?<br>• Spreekt het bericht dat zich focust op de gezondheidsvoordelen van bewegen u meer aan, of juist het bericht die meer de focus legt op het hebben van plezier en het bevorderen van sociaal contact? Wat is de reden dat u dat denkt?<br>• Is beweeggedrag volgens u het belangrijkst of zijn er andere behoeften die meer prioriteit hebben om aangepakt te worden bij 50-plussers?
**2C**	**Stijl**	Perceptie van 50-plussers over de stijl van het wervingsbericht voor deelname aan een beweegprogramma.	Inclusie<br>-Uitspraken over verschillende berichtvariabelen die ervoor zorgen dat het bericht overtuigingskracht heeft. Zoals feitelijke voorbeelden vs. abstracte, letterlijke vs. figuurlijke taal, humor vs. serieus/autoritair, oftewel de manier waarop het bericht gepresenteerd wordt met de gebruikte gedragsveranderingsmethodiek<br>-Uitspraken over de gewenste moeilijkheidsgraad/leesbaarheid van het wervingsbericht en de gebruikte termen, staat er teveel vakjargon in of is het aan de andere kant te kinderachtig?	• Wat is uw eerste indruk van deze berichten?<br>• Hoe kunnen we het ‘bewegen’ nu het beste noemen? Moeten we het sporten noemen, moeten we het bewegen noemen? Of nog iets anders?<br>• Wat vindt u van de leesbaarheid van het bericht? Denkt u dat dit iets is wat iedereen goed begrijpt? Is het voor iedereen goed te lezen?

			-Uitspraken over de lengte van de zinnen<br>-Uitspraken over de huidige of gewenste opmaak van het bericht<br>-Uitspraken over woorden en termen die de doelgroep wel of niet aanspreekt<br>-Opmerkingen over stijlfiguren die het bericht sterker of levendiger maken<br><br><u>Exclusie:</u><br>-Uitspraken die niet over de berichtvariabelen gaan maar over de inhoudelijke argumenten van het bericht, deze hebben ook meer te maken met emotie en beleving van de lezer -> **2B Soort argument**	• Bij berichten waarmee 50-plussers geïnformeerd worden over beweegprogramma's zijn vaak ter illustratie enkele foto's te zien. Op de slide ziet u een aantal foto's. Welk van deze foto's zou u het meest aanspreken? En wat is de reden dat deze u aanspreekt? En waarom andere juist niet?
**2D**	**Herhaling**	Perceptie van 50-plussers over de herhaling van het wervingsbericht	<u>Inclusie:</u><br>-Positieve en negatieve uitspraken over hoe vaak iemand het bericht onder ogen zou willen krijgen<br>-<br><br><u>Exclusie:</u><br>-Uitspraken over de verschillende kanalen of bronnen.	

1c Welke bron of combinatie van bronnen spreekt de doelgroep het meest aan

3. **Inputfactoren :Bron**

**Code**	**Input/ Communicatievariabele**	Definitie	Instructie voor codering	Voorbeeldvragen
 <br>**3A1**<br>**3A2**<br>**3A3**<br><br><br><br>**3A4**<br>**3A5**<br>**3A6**<br>**3A7**	**Soort Bron:**<br><br>**Gemeente**<br>**Huisarts**<br>**Andere Zorgprofessional:**<br>Sociale buurtteams, fysiotherapeuten, buurtsportcoaches<br>**Familielid**<br>**Vriend**<br>**Buurtgenoot**<br>**Overig**	De perceptie van 50-plussers m.b.t. de bronnen die hen juist wel of niet kunnen benaderen om mee te doen aan een beweegprogramma.	Inclusie:<br>- Uitspraken over bronnen die de doelgroep juist wel of niet moeten benaderen.<br><br>Exclusie:<br>-Uitspraken over de eigenschappen van bronnen -> **Bron eigenschappen 3B**<br>-Uitspraken over combinaties van bronnen -> **Bron aantal 3A1**	• Er zijn verschillende organisaties en personen die u kunnen wijzen op de mogelijkheid om mee te doen aan een bepaald beweegprogramma. Wie maakt de meeste kans om u te overtuigen hier aan mee te doen?<br>• Zijn er organisaties of personen die u absoluut niet moet benaderen voor een beweegprogramma?<br>• Zou je het bericht graag met iemand anders willen bespreken voordat je ja zegt? En wie zou dat dan moeten zijn?<br>• Hoe kijkt u ertegenaan als u op een

				beweegprogramma wordt gewezen door uw gemeente? En moet dit dan afkomstig zijn van een bepaald persoon binnen uw gemeente, bijvoorbeeld de wethouder, of maakt dat niet uit?<br>• Hoe kijkt u ertegenaan als u op een beweegprogramma wordt gewezen door uw gemeente? En moet dit dan afkomstig zijn van een bepaald persoon binnen uw gemeente, bijvoorbeeld de wethouder, of maakt dat niet uit?<br>• Of door uw huisarts of een andere zorgprofessional?<br>• Of juist een familielid, vriend of buurtgenoot?

				Of iemand die bekend is, -lokaal of landelijk? Zo ja, wie zou dit dan kunnen zijn?
**3B**	**Bron Aantal**	De perceptie van 50-plussers m.b.t. het aantal en de combinatie van bronnen die hen kunnen benaderen om mee te doen aan een beweegprogramma.	Inclusie:<br>-Uitspraken over de voorkeur, of juist niet, om door meerdere bronnen benaderd te worden voor een beweegprogramma<br>-Uitspraken over specifieke combinaties van bronnen<br>-Uitspraken over hoe verschillende bronnen verschillende kanten van het voordeel van bewegen kunnen belichten	• Hoe belangrijk is het voor u dat u niet uit één bron maar van meerdere verschillende personen/organisaties iets over het beweegprogramma hoort?
**3C**	**Bron eigenschappen:**	De perceptie van 50-plussers m.b.t. de eigenschappen van de bronnen die hen juist wel of niet kunnen benaderen om mee te doen aan een beweegprogramma.	Inclusie:<br>-Uitspraken over eigenschappen die niet onder 3C1 t/m 3C5 vallen	• Wat is de reden dat u wel of niet door een bepaalde bron benaderd wilt worden?
**3C1**	**Geloofwaardigheid**	De perceptie van 50-plussers over de geloofwaardigheid van bronnen die hun kunnen benaderen.	Inclusie:<br>-Uitspraken over bronnen waarvan de 50-plussers vinden dat zij expertise bezitten<br>-Uitspraken over bronnen waarvan de 50-plussers vinden dat ze betrouwbaar zijn<br>-Uitspraken over bronnen die volgens de doelgroep voldoende kennis bezitten om hen over dit onderwerp te benaderen	

**3C2**	**Aantrekkelijkheid**	De perceptie van 50-plussers over de aantrekkelijkheid van bronnen die hun kunnen benaderen.	Inclusie:<br>-Uitspraken over de aangenaamheid ofwel vriendelijkheid van de bron<br>-Uitspraken over de schoonheid van de bron, zoals iemand die zelf fit is<br>-Uitspraken over de bekendheid van de bron<br>-Uitspraken over de eigen overeenkomsten met de bron, zoals bij een vriend of vriendin het geval kan zijn	
**3C3**	**Macht**	De perceptie van 50-plussers over de (positieve) macht van bronnen die hun kunnen benaderen.	Inclusie:<br>-Uitspraken over bepaalde beloningen of straffen die een bron kan geven<br>-Uitspraken over bronnen die de doelgroep bepaald gedrag kan opleggen, bijvoorbeeld een huisarts of familielid<br>-Uitspraken over bronnen die het beweeggedrag kan controleren, zoals bijvoorbeeld een personal trainer	
**3C4**	**Leeftijd**	De perceptie van 50-plussers over de leeftijd van bronnen die hun kunnen benaderen		
**3C5**	**Geslacht**	De perceptie van 50-plussers van het geslacht van bronnen die hun kunnen benaderen		

1d. Welk kanaal of combinatie van kanalen spreekt de doelgroep het meest aan

Inputfactoren 4: Kanaal

Code	Input/ Communicatievariabele	Definitie	Instructie voor codering	Voorbeeld vragen
	**Soort Kanaal:**	De perceptie van 50-plussers m.b.t. het kanaal/medium die hen juist wel of niet aanspreekt om mee te doen aan een beweegprogramma	<u>Inclusie:</u><br>-Uitspraken over de soort kanalen die de beweeginterventie onder de aandacht kunnen brengen bij de doelgroep.<br><br><u>Exclusie:</u><br>-Uitspraken over specifieke eigenschappen van kanalen -> **Kanaal eigenschappen 4B**<br>-Uitspraken over het aantal en de combinatie van kanalen. -> **Kanaal Aantal 4C**	• Wat is voor u een goede manier om informatie over een beweegprogramma te ontvangen?<br>• Denkt u dat er ook manieren zijn om de informatie te verspreiden die juist helemaal niet effectief is of zelfs averechts zou kunnen werken?<br>• Wat is de reden (dat u wel of niet het bericht door een bepaald kanaal onder de aandacht gezien wilt worden)?
**4A1**	**Massamedia**	De perceptie van 50-plussers m.b.t. de benadering van een beweegprogramma via massamedia.	<u>Inclusie:</u><br>-Uitspraken over luister en kijktijd<br>-Uitspraken over specifieke televisie en radio zenders<br>-Uitspraken over reclame spotjes op televisie en radio en wanneer worden deze als leuk ervaren en wanneer als ergerlijk<br>-Uitspraken over de voorkeur voor een programma of reclameblok waarin een beweeginterventie aan bod kan komen	• Wat zou u van een bericht via de lokale radio vinden? En zo ja, welke lokale radio zender dan?<br>• En de televisie?

**4A2**	**Online media**	De perceptie van 50-plussers m.b.t. de benadering van een beweegprogramma via online media.	Inclusie:<br>-Uitspraken over social media<br>-Uitspraken over websites<br>-Uitspraken over e-mail	• Of via social media? En welke social media denkt u dan aan?<br>• Zou u liever een e-mail ontvangen? Wat is de reden dat u dat vindt?
**4A3**	**Papieren media**	De perceptie van 50-plussers m.b.t. de benadering van een beweegprogramma via papieren media.	Inclusie:<br>-Uitspraken over huis aan huis bladen<br>-Uitspraken over kranten en tijdschriften<br>-Uitspraken over geadresseerde brieven	• Wat zou u ervan vinden om een brief thuis te krijgen met informatie over het beweegprogramma? Wat is de reden dat u dat vindt?<br>• Zou een artikel in de krant of tijdschrift of in het lokale krantje van de gemeente of de wijk die u leest uw aandacht trekken?<br>• Zou een folder over een beweegprogramma uw aandacht kunnen trekken? Waarom denkt u?
**4A4**	**Mond tot mond reclame**	De perceptie van 50-plussers m.b.t. de benadering van een beweegprogramma via mond tot mond reclame.	Inclusie:<br>-Uitspraken over directe benadering d.m.v. mond tot mond reclame<br>-Uitspraken over posters en flyers op plekken waar de doelgroep samenkomt<br>-Uitspraken over locaties waar de doelgroep samenkomt als werk, kerk, buurthuizen etc.	• Zou een poster uw aandacht kunnen trekken? Waarom denkt u? En waar zou zo'n poster dan moeten hangen?<br>• We hebben nu een aantal manier besproken waarom u informatie zou kunnen krijgen over een beweegprogramma: En moet dat dan een bericht zijn van iemand die lokaal

				bekend is? Of mag dat ook een algemeen iemand zijn?<br>• Of via persoonlijk contact: Hoe zou u het vinden als echt een persoon (huisarts, fysio, sociaal werker) u fysiek informeert over het beweegprogramma?
**4B**	**Aantal**	De perceptie van 50-plussers m.b.t. het aantal en de combinatie van kanalen/media die hen kunnen benaderen om mee te doen aan een beweegprogramma.	Inclusie:<br>-Uitspraken over de voorkeur, of juist niet, om door meerdere kanalen benaderd te worden voor een beweegprogramma.<br>-Uitspraken over specifieke combinaties van kanalen.	• Denkt u dat u eerder geneigd zou zijn om mee te doen aan een beweegprogramma wanneer u bijvoorbeeld een brief thuis gestuurd krijgt over het beweegprogramma, vervolgens een radiospotje hoort en daarna ook nog een poster ziet op een plek waar u regelmatig komt? Waarom denkt u dit?
	**Kanaal eigenschappen:**	De perceptie van 50-plussers m.b.t. de eigenschappen van de kanalen die hen juist wel of niet kunnen benaderen om mee te doen aan een beweegprogramma		• Wat is de reden dat u wel of niet het bericht door een bepaald kanaal onder de aandacht gebracht wil worden?
**4C1**	**Audio/Visueel Geschreven tekst/gesproken tekst**	De perceptie van 50-plussers m.b.t. het gebruik binnen de kanalen van gesproken c.q. geschreven tekst	Inclusie;<br>-Uitspraken over het gebruik van geschreven c.q gesproken tekst binnen de kanalen	
**4C2**	**Context**	De perceptie van 50-plussers m.b.t. de context waarbinnen	Inclusie	

		de communicatie via de kanalen plaatsvindt	-Uitspraken over de context waarbinnen de communicatie binnen de kanalen plaats vindt:<br>-1 persoon of gezelschap met meerdere mensen<br>-Gezellige of formele sfeer	
**4C3**	**De manier waarop de boodschap non-verbaal wordt gebracht**	De perceptie van 50-plussers m.b.t. het gebruik van non-verbale communicatie binnen de kanalen	Inclusie:<br>-Uitspraken m.b.t. de non-verbale communicatie binnen de kanalen:<br>-Met welke gezichtsuitdrukking, houding, spraakgebruik	

1 e Wat zijn volgens de doelgroep faciliterende en belemmerende determinanten m.b.t. het doelgedrag c.q. het aanmelden en meedoen aan de beweeginterventie Actief Plus hoe kunnen de belemmerende determinanten zo goed mogelijk verholpen worden?

5. Inputfactoren: Doelgedrag c.q. het aanmelden voor Actief Plus (registratieproces) en het meedoen aan Actief Plus

M.b.t. het aanmelden voor Actief Plus

Code	Determinant/Input/ Communicatievariabele	Definitie	Instructie voor codering	Voorbeeldvragen
**5A**	**Onmiddelijkheid**	De perceptie van 50-plussers m.b.t. de behoefte aan onmiddellijke actie om mee te doen aan een beweegprogramma.	Inclusie:<br>-Uitspraken over het onmiddellijk of vertraagd kunnen starten van een beweegprogramma<br>-Uitspraken over of de doelgroep de te nemen stappen voordat men kan beginnen met Actief Plus de moeite waard vind<br>-Uitspraken over de tijd die zit tussen het ontvangen van het wervingsbericht en het starten met Actief Plus, zou de doelgroep nog steeds mee willen doen als er niet direct gestart kan worden?	• Ziet u het als een drempel wanneer u zich eerst moet aanmelden voor een beweegprogramma in plaats van dat u direct kunt starten? (Bijvoorbeeld door direct een online advies te krijgen zonder aanmelding vooraf, of door op een locatie gewoon binnen te lopen voor adviezen, zonder

				u vooraf te moeten melden.) Wat is de reden dat u dat vindt?<br>• Soms kan het enkele dagen duren tot uw aanmelding verwerkt is, en voordat u daadwerkelijk kunt starten met het programma. Zou dat voor u een bezwaar zijn?
**5B**	**Uitkomstverwachting**	De perceptie van de 50-plussers m.b.t de uitkomstverwachting over het aanmeldproces	-Uitspraken over de verwachting van de uitkomsten van het aanmeldproces	

M.b.t het meedoen aan Actief Plus

Code	Determinant/Input/ Communicatievariabele	Definitie	Instructie voor codering	Voorbeeld vragen
**6A**	**Overtuigende impact**	De perceptie van 50-plussers m.b.t. de overtuigende impact van de Actief Plus interventie.	<u>Inclusie:</u><br>-Uitspraken over de directe motivatie om mee te doen aan de Actief Plus interventie<br>-Uitspraken over hoe snel de doelgroep het wervingsbericht van de Actief Plus interventie denkt te vergeten	• Als u dit zo hoort, lijkt dit u dan een programma waar u aan mee zou willen doen?<br>• Wanneer de vragenlijst is ingevuld krijgt u dus een uitgebreid advies over hoe u meer kunt bewegen volledig afgestemd op uw eigen situatie. Ziet u voor of nadelen aan deze vorm van een beweegprogramma?

				• Wat zouden voor u overwegingen zijn om mee te doen aan een beweegprogramma?
**6B**	**Relatief voordeel**	De perceptie van de 50-plussers over het voordeel van de Actief Plus interventie t.o.v. geen of een alternatieve interventie.	Inclusie:<br>-Uitspraken die aangeven dat de Actief Plus interventie beter of slechter is dan geen of een andere interventie<br>-Uitspraken over kenmerken van de Actief Plus interventie die hem wel of niet aantrekkelijker maakt dan andere interventies. Bijvoorbeeld omdat het:<br>-een advies op maat interventie is<br>-een online interventie is	• En wat zijn redenen die ervoor zorgen dat u niet wilt meedoen aan een beweegprogramma?<br>• Er is niet iemand die de resultaten face-to-face met u bespreekt, wat vindt u hiervan?<br>• Of u de adviezen opvolgt is uw eigen verantwoordelijkheid. Wat vindt u hiervan?<br>• Wat zijn volgens u verder nog voordelen of nadelen van dit beweegprogramma? En wat is de reden dat u dat vindt?
**6C**	**Mogelijkheid tot uitproberen**	De perceptie van de 50-plussers over de mogelijkheid om Actief Plus uit te proberen en hier weer mee te kunnen stoppen.	Inclusie:<br>-Uitspraken over de wens om meer over Actief Plus te leren alvorens zich aan de interventie te committeren<br>-Uitspraken over de wens om Actief Plus te kunnen stoppen of pauzeren	• En wat zijn redenen die ervoor zorgen dat u niet wilt meedoen aan een beweegprogramma?
**6D**	**Complexiteit**	De perceptie van 50-plussers over de moeilijkheidsgraad van het meedoen aan Actief Plus.	Inclusie:<br>-Uitspraken over de ingewikkeldheid van Actief Plus<br>-Uitspraken over de duur van Actief Plus	• En wat zijn redenen die ervoor zorgen dat u niet wilt meedoen aan een beweegprogramma?

			-Uitspraken over de te nemen stappen bij aanmelding bij Actief Plus	• Voor het maken van een gedegen persoonlijk advies zoals dat gebeurt in Actief Plus, is het invullen van een lange vragenlijst noodzakelijk. Het invullen van deze vragenlijst duurt ongeveer 45 minuten. Hoe kijkt u hier tegen aan? En maakt het voor u een verschil of u deze vragenlijst online of op papier zou invullen?
**6E**	**Compatibiliteit**	De perceptie van de 50-plussers over de mate waarin de Actief Plus interventie aansluit bij hun dagelijks leven, doelen, normen en waarden	<u>Inclusie:</u><br>-Uitspraken over de mate van aansluiting van de Actief Plus interventie in het huidig dagelijks leven<br>-Uitspraken over de mate van aansluiting van de Actief Plus interventie bij de eigen doelen, normen en waarden	• En wat zijn redenen die ervoor zorgen dat u niet wilt meedoen aan een beweegprogramma?<br>• Omdat het advies op de persoon afgestemd wordt kan iedereen op zijn of haar eigen manier meer gaan bewegen. Wat vindt u hiervan ten opzichte van een beweegprogramma waarbij iedereen hetzelfde doet en hetzelfde doel heeft?
**6F**	**Uitkomstverwachting**	De perceptie van de 50-plussers m.b.t. de kwaliteit en validiteit van Actief Plus ter ondersteuning van de overtuiging dat Actief Plus zal	<u>Inclusie:</u><br>-Uitspraken die betrekking hebben op het bewustzijn van de sterkte en kwaliteit van Actief Plus. Zoals het is wetenschappelijk ontwikkeld dus ik heb er vertrouwen in, of juist niet	• En wat zijn redenen die ervoor zorgen dat u niet wilt meedoen aan een beweegprogramma?

		leiden tot gewenste uitkomsten.	-Uitspraken die betrekking hebben op het ontbreken van bewijs van de werking van Actief Plus<br>-Uitspraken over het verwachte effect op het eigen beweeggedrag door Actief Plus<br>-Uitspraken over het verwachte effect op andere vlakken dan beweeggedrag, bijvoorbeeld op het gebied van positieve gezondheid, eenzaamheid, gezelligheid, groepsvorming etc.<br>-Uitspraken over ervaringen met andere interventies en hoe die de verwachtingen van Actief Plus beïnvloeden<br><br>Exclusie:<br>-Uitspraken die niet gaan over de verwachtingen van Actief Plus maar de behoeften van de respondent	

In de vorige versie hebben jullie aangegeven dat Sociaal contact beter onder doelgedrag zou kunnen vallen dan onder 1. Inputfactoren : doelgroep/ontvanger. Betekent dat we het kunnen onder meedoen aan Actief Plus of onder aanmelden voor Actief Plus?

**6G**	**Sociaal contact**	De perceptie van 50-plussers over de bevorderende en/of belemmerende werking van hun sociale contacten wanneer het gaat om het deelnemen aan een beweegprogramma	Inclusie:<br>- Inclusie:<br>-Uitspraken over het wel of niet hebben van sociaal contact<br>-Uitspraken waarin de persoon aangeeft dat sociaal contact hem of haar helpt of belemmerd om voldoende te bewegen	Zou je het bericht graag met iemand bespreken voordat je ja zegt? En wie zou dat moeten zijn? Welke persoon of organisatie?<br>Op welke manier zouden wij deelname aan het beweegprogramma voor u makkelijker kunnen maken?

				-Is er bijvoorbeeld een hulplijn nodig?<br>-Niet iedereen is even handig met computers: zou er hulp nodig zijn om de vragenlijst online in te vullen, bijvoorbeeld maatschappelijk werk o.i.d.
