# Supplementary material for: “I Don’t Believe in Age; I Believe in Staying Enthusiastic”: An Exploratory Qualitative Study into Recruitment Strategies Stimulating Middle-Aged and Older Adults to Join Physical Activity Interventions
Source: Geriatrics (Basel). 2024 Jun 13;9(3):80. doi: 10.3390/geriatrics9030080 (PMC11202473; doi:10.3390/geriatrics9030080)
Supplement: Supplementary file 1 [file geriatrics-09-00080-s001.zip › 21032024_File_S1 Interview Guide.pdf]

Interviewhandleiding Individuele Interviews

Vooraf

- Opname starten
- Checken dat informed consent ondertekend retour is

Introductie van de bijeenkomst (5 minuten)

- Welkom heten

  Welkom, dank dat je tijd had voor dit gesprek. Mijn naam is xxxxxx en ik ben *functie* bij de Open Universiteit. Zoals al in de uitnodigingsmail vermeld, nemen we dit gesprek op. Het wordt dan later uitgetypt zoals we in de informatiebrief al aangeven. Dus voor ik het vergeet start ik nu de opname. We noteren ook altijd graag wat achtergrond gegevens daarom zou ik je graag nog even willen vragen om nog even aan te geven wat je leeftijd is, wat je opleidingsniveau is en of je wel of niet een actieve beweger bent of bent geweest.

  Korte uitleg doel van het interview

  Ik zal nog even een korte uitleg geven. Bijna iedereen weet dat bewegen goed voor je is. Toch zijn er veel 50-plussers die niet zo veel bewegen als dat van belang is voor een goede gezondheid en voor welzijn, lekker in je vel te zitten. Onder vijftigplus verstaan we trouwens iedereen ouder dan 50 dus ook tachtigers bijvoorbeeld. Om ervoor te zorgen dat er toch voldoende bewogen wordt zijn er beweeginterventies ontwikkeld, onder andere door de Open Universiteit. Dit zijn programma’s die mensen helpen om voldoende te bewegen. Er zijn echter maar weinig 50-plussers die gebruik maken van deze programma’s. We willen graag met u in gesprek gaan om er achter te komen hoe dat komt. In dit gesprek willen we het dan ook met u hebben over het bevorderen van beweeggedrag, hoe belangrijk dat volgens u is en waarom. En wat volgens u de beste manier is om enthousiast gemaakt te worden om mee te doen aan een beweeginterventie. Ons uiteindelijk doel is dat de beweegprogramma’s ook daadwerkelijk worden gebruikt door de doelgroep, dus 50 plussers.
- Structuur van het gesprek:

  Wij hebben verschillende gespreksonderwerpen en dat zijn heel kort de doelgroep, de beweeginterventie Actief Plus, het bericht waarmee mensen worden geïnformeerd over Actief Plus, de boodschap die daarin staat, het kanaal waarmee we mensen proberen te bereiken, en het doelgedrag. We leggen straks natuurlijk bij elk onderwerp uit wat het precies is.
- Ons gesprek duurt ongeveer 1,5 uur.
- Heeft u nog vragen voordat we beginnen met het gesprek?

Doelgroep

(Achtergrondinformatie voor interviewer: theorie van McGuire benoemt hier o.a. leeftijd, opleidingsniveau, geslacht, etniciteit, persoonlijkheid, leefstijl, fysieke beperkingen, gezondheidsvaardigheden, eigen effectiviteit, beweegkapitaal etc een rol kunnen spelen).

We hebben het vandaag over beweegprogramma's. Voordat we daar over gaan praten even een paar wat meer algemene vragen:

1. Vind je het belangrijk dat het beweeggedrag van 50-plussers wordt gestimuleerd? Wat zijn de redenen dat je dat belangrijk vindt of juist niet belangrijk vindt?
2. Voel jij je aangesproken als het gaat over 50-plussers? Zie je jezelf als een 50-plusser? Tot welke groep zie je je wel? Vindt je het aansprekender als het gaat over een brede doelgroep qua leeftijd voor beweegprogramma's of zou je het liever wat meer gericht op bepaalde leeftijdscategorieën zien?
3. Is beweeggedrag volgens u het belangrijkst of zijn er andere behoeften die meer prioriteit hebben om aangepakt te worden bij 50-plussers?
    - *Bijvoorbeeld behoefte aan sociaal contact, plezier in het leven, onafhankelijkheid.*

We gaan het nu hebben over beweegprogramma's. Beweegprogramma's zijn samenhangende gerichte georganiseerde activiteiten om mensen te stimuleren om meer te willen, kunnen en gaan bewegen. Zoals eerder aangegeven proberen beweegprogramma's te helpen om mensen meer te laten bewegen. De manier waarop zo'n beweegprogramma mensen stimuleert om te bewegen kan heel verschillend zijn. Zo kan een programma bijvoorbeeld bestaan uit het geven van informatie, zoals tips en adviezen voor beweegactiviteiten die u van uw huisarts of via een flyer krijgt, maar het kunnen ook een aantal lessen zijn bij een sportvereniging waarbij u extra intensief begeleid wordt of kennis maakt met verschillende sporten zodat u dit daarna zelfstandig voort kunt zetten.

4. Denkt u dat het noodzakelijk is dat er beweegprogramma's worden ingezet om het beweeggedrag van 50-plussers te bevorderen? Wat is de reden dan u dit vindt?
5. Wat zouden voor u overwegingen zijn om mee te doen aan een beweegprogramma?
    - *Bijvoorbeeld het opbouwen van conditie en kracht, minder kans om te vallen, vergroten zelfvertrouwen, langer zelfstandig kunnen zijn, spelen met kleinkinderen, welzijn verbeteren, zo lang mogelijk in staat blijven om dat te doen wat je leuk vind in het leven.*
6. En wat zijn redenen die ervoor zorgen dat u dat niet wilt meedoen aan een beweegprogramma?
    - *Bijvoorbeeld vanwege de leeftijd, vroeger beweeggedrag, geen tijd, bijkomende kosten, huidige fysieke of mentale belemmeringen.*

Dat waren de eerste vragen, dan gaan we nu naar het volgende onderwerp.

Actief Plus

We bespreken nu graag even een specifiek beweegprogramma, namelijk de Actief Plus interventie. Dit is een interventie die door de Open Universiteit ontwikkeld is, waardoor wij deze interventie natuurlijk goed kennen. In dit gesprek gebruiken we deze interventie puur even als voorbeeld.

Actief Plus is een programma waarin u advies krijgt over beweging en is ook geschikt voor mensen die een lichamelijke beperking hebben waardoor het moeilijk is om te bewegen.* U wordt door de gemeente uitgenodigd voor dit gratis programma. Deelname aan Actief Plus is heel eenvoudig. U vult een vragenlijst in, en krijgt vervolgens een beweegadvies dat op u is afgestemd. Indien u gezondheidsklachten heeft (bijvoorbeeld een evenwichtsstoornis, versleten heup of hartaandoening), dan wordt hiermee rekening gehouden. U krijgt tips over hoe u meer kunt bewegen en waar u dat kunt doen. U hoeft daarbij niet alleen aan sport te denken; bewegen in en rond het huis telt ook mee. Wij geven ook tips over hoe u voldoende kunt blijven bewegen. Actief Plus is dus geschikt voor mensen die weinig bewegen, maar ook voor mensen die veel bewegen. Voor de adviezen van Actief Plus hoeft u nergens heen: u ontvangt de op maat gemaakte adviezen gewoon thuis of per e-mail. "

1. Dat is in het kort de Actief Plus interventie, heb ik hem zo duidelijk uitgelegd of heeft u nog vragen?
2. Als u dit zo hoort, lijkt dit u dan een programma waar u aan mee zou willen doen? Wat is daar de reden voor?
3. Wanneer de vragenlijst is ingevuld krijgt u dus een uitgebreid advies over hoe u meer kunt gaan bewegen volledig afgestemd op uw eigen situatie. Ziet u voordelen of nadelen aan deze vorm van een beweegprogramma?

- Omdat het advies op de persoon afgestemd wordt kan iedereen op zijn of haar eigen manier meer gaan bewegen. Wat vindt u hiervan ten opzichte van een beweegprogramma waarbij iedereen hetzelfde doet en hetzelfde doel heeft? (*laagdrempelig, te individueel*).
- Voor het maken van een gedegen persoonlijk advies zoals dat gebeurt in Actief Plus, is het invullen van een lange vragenlijst noodzakelijk. Het invullen van deze vragenlijst [deelnemers vragenlijst laten zien] duurt ongeveer 15 minuten. Hoe kijkt u hier tegen aan? En maakt het voor u een verschil of u deze vragenlijst online of op papier zou invullen?
- Er is niet iemand die de resultaten face-to-face met u bespreekt, wat vindt u hiervan?
    - *(Mogelijk nadeel is dat er niemand is om vragen aan te stellen, voordeel is dat alles in eigen tijd en tempo gelezen kan worden).*
- Of u de adviezen opvolgt is uw eigen verantwoordelijkheid. Wat vindt u hiervan?
    - *Mogelijk voordeel is niet gepusht worden, dit werkt bij sommigen averechts. Is tegelijkertijd een nadeel voor de mensen die wel een stok achter de deur nodig hebben.*

4. Wat zijn volgens u verder nog voordelen of nadelen van dit beweegprogramma? En wat is de reden dat u dat vindt?
    - *Bijvoorbeeld compleet toegespitst op de persoon, laagdrempelig, snel advies of uit geen face-to-face contact om de resultaten te bespreken, wel advies maar geen concrete beweegactiviteiten, je moet zelf wat met de adviezen gaan doen.*

Dankjewel voor je antwoorden, heel bruikbaar, dan gaan we nu naar het volgende onderwerp.

Bericht

(Achtergrondinformatie voor interviewer: vragen stellen en doorvragen met theorie van McGuire in gedachten zijnde dat hier de stijl, taalgebruik, behoeften (fysieke kracht, minder eenzaamheid, welzijn, sociale contacten etc), lengte van het bericht, vorm, uiterlijk, dingen die opvallenderwijs juist wel of niet zijn opgenomen, etc een rol kunnen spelen).

Waar we het nu met jullie over willen hebben: Hoe kunnen we u het beste op de hoogte stellen/informeren over beweegprogramma's?. Wanneer u voor het eerst in aanraking komt met een beweegprogramma zal dit door middel van een bepaald bericht zijn, bijvoorbeeld in de vorm van een folder of een advertentie. Dit bericht heeft dan het doel om uw interesse te wekken en hopelijk over te halen om mee te doen aan het programma. Wij willen dit bericht zo goed mogelijk afstemmen op wat bij jou als 50-plussers past. Hierbij zijn wij benieuwd wat u juist wel of niet aanspreekt wanneer het gaat om de inhoud van zo'n bericht. Om hier een beter beeld bij te krijgen hebben wij twee korte voorbeelden gemaakt van hoe zo'n bericht eruit zou kunnen zien. Ik zal deze laten zien nu.
[Voorbeelden op sheet laten zien: betreft: Flyer_deelnemers_tbvFocusgroep.pdf]

1. Wat is uw eerste indruk van deze berichten?
    - Zijn er woorden die u aanspreken of juist niet? Wat is de reden dat u dat vindt?
    - Wat vindt u van de term 50-plusser? Wat zou een betere term zijn?
    - Hoe kunnen we het 'bewegen' nu het beste noemen? Moeten we het sporten noemen, moeten we het bewegen noemen, of actief zijn? Of nog iets anders?
    - Denkt u dat het beter is om te vertellen dat je van meer bewegen een betere conditie en meer kracht krijgt, of juist dat je door meer bewegen onafhankelijker in het leven kan staan, dat je cognitie op pijl blijft, dan je langer kan blijven doen wat je leuk vindt om te doen, dat je anderen kan ontmoeten? Wat maakt dat u dit vindt?
    - Wat vindt u van de leesbaarheid van het bericht? Denkt u dat dit iets is wat iedereen goed begrijpt?
    - Hoe aansprekend/motiverend vind je de berichten?
2. Welke informatie vindt u interessanter: "" Voldoende bewegen werkt beschermend tegen het ontwikkelen van verschillende ziekten als hart- en vaatziekten, beroerte, diabetes en verschillende vormen van kanker." Of: "Voldoende bewegen heeft een positief op de mentale gezondheid waarbij het gevoel van welzijn en kwaliteit van leven wordt vergroot, daarnaast kan voldoende bewegen het ontwikkelen van een depressie voorkomen". Wat is de reden dat u dat vindt?
3. Spreekt het bericht dat zich focust op de gezondheidsvoordelen van bewegen u meer aan, of juist het bericht die meer de focus legt op het hebben van plezier en het bevorderen van sociaal contact? Wat is de reden dat u dat denkt?
4. Zijn er zaken die niet in de berichten staan maar waarvan u wel vindt dat ze benoemd moeten worden? Welke zijn dit?
5. Bij berichten waarmee 50-plussers geïnformeerd worden over beweegprogramma's zijn vaak ter illustratie enkele foto's te zien. Op de slide ziet u een aantal foto's. [Slide met foto's laten zien], dus printen of scherm delen. Welk van deze foto's zou u het meest aanspreken? En wat

is de reden dat deze u aanspreekt? En waarom andere juist niet? (*te nederlands/buitenlands, te veel/weinig nadruk op bewegen, verkeerde/juiste leeftijdsgroep, te zomers/winters, iets neutraals als afbeelding van wandelschoen beter/slechter etc)*

Dat waren de vragen voor het onderwerp Bericht, dan gaan we nu naar de volgende set vragen en die gaat over de bron, dus wie informeert jou over een beweegprogramma.

Bron

(Achtergrondinformatie voor interviewer: (door)vragen met theorie van McGuire in gedachten zijnde dat hier vertrouwen in de bron, eigenschappen van de bron (wie, welke instelling, geslacht of leeftijd van persoon etc een rol kunnen spelen), waargenomen deskundigheid etc een rol spelen.

1. Er zijn verschillende organisaties en personen die u kunnen wijzen op de mogelijkheid om mee te doen aan een bepaald beweegprogramma. Wie maakt de meeste kans om u te overtuigen hier aan mee te doen?
    - Hoe kijkt u er tegenaan als u hierop gewezen wordt door uw gemeente?(*wekt vertrouwen of juist betuttelend)* En moet dit dan afkomstig zijn van een bepaalde persoon binnen uw gemeente, bijv de wethouder, of maakt dat niet uit?
    - Of door uw huisarts of een andere zorgprofessional?
        - *Bijvoorbeeld sociale buurtteams, fysiotherapeuten, buurtsportcoaches etc.*
    - Of juist een familielid, vriend of buurtgenoot?
    - Of iemand die bekend is,- lokaal of landelijk? Zo ja, wie zou dit dan kunnen zijn?
    - Wie van deze zou uw voorkeur hebben? Waarom is dat volgens u zo?
        - *Bijvoorbeeld een gemeente of zorgprofessional kan expertise uitstralen en daardoor betrouwbaar overkomen. Een familielid, vriend of buurtgenoot kan als leuk of sociaal aantrekkelijk worden gezien, iemand waar aan gespiegeld kan worden of die je sportmaatje kan zijn.*
    - Hoe kijkt u er tegenaan wanneer een universiteit u wijst op een beweegprogramma?
    - Is er nog iemand anders of een andere organisatie waarvan u het prettig zou vinden dat hij of zij u op een dergelijk beweegprogramma wijst?
2. Zijn er ook organisaties of personen die u juist absoluut niet moeten benaderen voor een beweegprogramma? Waarom niet?
        - *Bijvoorbeeld opgevat als bemoeienis of als te commercieel. Evt bespreken publiek vs commercieel, peers vs professional vs bekend persoon.*
3. Hoe belangrijk is het voor u dat u niet uit 1 bron maar van meerdere verschillende personen/organisaties iets over het beweegprogramma hoort?
4. Zou je het bericht graag met iemand anders willen bespreken voordat je JA zegt? En wie zou dat dan moeten zijn? Welke persoon of organisatie? (*suggestie: huisarts, fysiotherapeut, leeftijdsgenoot, familie)*

Dat was het dan voor dit onderdeel: allemaal erg nuttige informatie voor ons.

Kanaal

(Achtergrondinformatie voor interviewer: vragen stellen en doorvragen met theorie van McGuire in gedachten zijnde dat hier gesproken/geschreven tekst, audio/visueel, woord of beeld, context, online of papier etc , internetvaardigheid, soort media etc, een rol kunnen spelen).

Dan wil ik nu graag verdergaan op het volgende onderdeel en overgaan op de manier waarop jullie graag de informatie over een beweegprogramma willen ontvangen. Zo kunt u deze bijvoorbeeld lezen in een folder, een reclame via het internet of het horen via de radio enzovoorts. Het verschil met het vorige onderdeel zit het hem erin dat het nu niet gaat om de organisatie of persoon, maar de manier waarop, via welk kanaal als het ware.

1. Wat is voor u een goede manier om die informatie over een beweegprogramma te ontvangen? we zullen een aantal voorbeelden noemen.
    - Wat zou u ervan vinden om een brief thuis te krijgen met informatie over het beweegprogramma? Wat is de reden dat u dat vindt?
    - Of zou u liever een e-mail ontvangen? Wat is de reden dat u dat vindt?
    - Zou een folder over een beweegprogramma uw aandacht kunnen trekken? Waarom denkt u? En waar zou die folder dan moeten liggen? Of in je brievenbus komen?
    - Zou een poster uw aandacht kunnen trekken? Waarom denkt u? Een waar zou zo’n poster dan moeten hangen?
    - Zou een artikel in de krant of tijdschrift of in het lokale krantje van de gemeente of de wijk die u leest uw aandacht trekken? Wat is de reden dat u dat denkt?
    - Wat zou u van een bericht via de lokale radio vinden? En zo ja, welke lokale radio zender dan?
    - En de televisie?
    - Of via social media? En welke social media denk je dan aan? Facebook, Instagram, LinkedIn bijvoorbeeld.
    - We hebben nu een aantal manier besproken op welke manier u informatie zou kunnen krijgen over een beweegprogramma: En moet dat dan een bericht zijn van iemand die lokaal bekend is? Of mag dat ook een algemeen iemand zijn?
    - Of via persoonlijk contact: Hoe zou u het vinden als echt een persoon (huisarts, fysio, sociaal werker) u fysiek informeert over het beweegprogramma?
2. Indien in het voorgaande nog niet besproken: Denkt u dat er ook manieren zijn om de informatie te verspreiden die juist helemaal niet effectief is of zelfs averechts zou kunnen werken? Welke zijn dit volgens u?
3. Denkt u dat u eerder geneigd zou zijn om mee te doen aan het beweegprogramma wanneer u bijvoorbeeld een brief thuis gestuurd krijgt over het beweegprogramma, vervolgens een radio spotje hoort en daarna ook nog een poster ziet op een plek waar u regelmatig komt? Waarom denkt u dit? Wat zou een goede combinatie zijn?

Doelgedrag

Dan wil ik nu verdergaan op het laatste onderdeel. Stel, u heeft op wat voor manier dan ook informatie ontvangen over een beweegprogramma. U denkt bij uzelf wat een goed initiatief, ik wil zeker meer bewegen en ik ga meedoen! Dan willen wij er graag achter komen hoe jullie aankijken tegen de te nemen stappen die je moet zetten om ook echt te kunnen starten en hoe wij daarin kunnen ondersteunen. *(als iemand aangeeft absoluut niet aan programma's mee te willen doen, toch proberen om deze vragen te laten beantwoorden, want ook mensen die niet mee willen doen kunnen een mening hebben hierover die voor ons bruikbaar is).*

1. De te ondernemen stappen zullen verschillen per beweegprogramma, en kunnen ook afhankelijk zijn van de manier waarop u de eerdere informatie ontvangen hebt: we zullen de stappen doorspreken die genomen zouden moeten worden voor deelname aan de eerder besproken Actief Plus interventie. We gaan die nu even doorlopen: hier sheet 19 laten zien uit presentatie en ook voorbeeld antwoordkaart of voorbeeld inlognaambrief.
2. Voor Actief Plus heb je een inlogcode en wachtwoord nodig als je de vragenlijst online wil invullen, en als je op papier wilt invullen, dan moet je een papieren vragenlijst opvragen. Dit kan bijvoorbeeld telefonisch, per mail, via een website, of door het terugsturen van een antwoordkaart. Hoe kijkt u hier tegen aan? Welke manier van aanmelden heeft u voorkeur?
3. Als je hoort of leest over Actief Plus via een folder of poster, dan moet je dus zelf nog iets doen: een telefoonnummer bellen of naar een website gaan. Is dat okay of juist bezwaarlijk als je dus dat nummer moet noteren of een foto van de link moet maken om je aan te melden?
4. Ziet u het als een drempel wanneer u zich eerst moet aanmelden voor een beweegprogramma in plaats van dat u direct kunt starten? (Bijvoorbeeld door direct een online advies te krijgen zonder aanmelding vooraf, of door op een locatie gewoon binnen te lopen voor adviezen, zonder u vooraf te moeten melden.) Wat is de reden dat u dat vindt?
5. Het kan enkele dagen duren tot uw aanmelding verwerkt is, en voordat u daadwerkelijk kunt starten met het programma. Zou dat voor u een bezwaar zijn?
6. Op welke manieren zouden wij deelname aan het beweegprogramma voor u makkelijker kunnen maken?
   - Is er bijvoorbeeld een hulplijn nodig? Fysiek, digitaal, telefonisch?
   - Niet iedereen is even handig met computers: zou er hulp nodig zijn om de vragenlijst online in te vullen, bijvoorbeeld maatschappelijk werk o.i.d.

Afsluiting

Dan zijn we aan het einde van de bijeenkomst gekomen. Ik wil u nogmaals hartelijk danken dat u met ons in gesprek wilde gaan. En ook voor de fijne manier waarop wij hebben kunnen spreken over dit onderwerp. Met uw antwoorden heeft u ons veel geholpen om beter te begrijpen hoe wij u kunnen benaderen. De resultaten van dit gesprek en nog een ander gesprek gebruiken wij om ervoor te zorgen dat een beweegprogramma als Actief Plus bij jullie onder de aandacht komt en hopelijk jullie interesse wekt om mee te doen aan het beweegprogramma.
